# Airway injury and pneumomediastinum associated with less invasive surfactant administration in a premature neonate: a case report

**DOI:** 10.1186/s12887-021-02981-w

**Published:** 2021-11-10

**Authors:** Talal Altamimi, Brooke Read, Orlando da Silva, Soume Bhattacharya

**Affiliations:** Neonatal - Perinatal Medicine, University of Western, 800 Commissioners Rd E, D4-200, London, Ontario N6A 5W9 Canada

**Keywords:** Less invasive surfactant administration, Minimal invasive surfactant therapy, Complications, Tracheal perforation, Air leak, Pneumomediastinum, Hobart method, Preterm, Case report

## Abstract

**Background:**

The use of less invasive surfactant administration (LISA)/minimally invasive surfactant therapy (MIST) has increased due to its potential advantage over traditional surfactant delivery methods through an endotracheal tube. Known complications for this procedure include failure of the first attempt at insertion, desaturation, and bradycardia. To the best of our knowledge, this is the first reported case of pneumomediastinum and subcutaneous emphysema following LISA.

**Case presentation:**

A preterm newborn born at 27 weeks of gestation presented with respiratory distress syndrome requiring surfactant replacement. LISA using the Hobart method was completed. There was a report of procedural difficulty related to increased resistance to insertion of the 16G angiocath. The newborn was subsequently noted to have subcutaneous emphysema over the anterior aspect of the neck and substantial pneumomediastinum on radiological assessment. Associated complications included hypotension requiring inotropic support. The newborn was successfully managed conservatively, with complete resolution of the air leak.

**Conclusions:**

Upper airway injury leading to air leak syndrome is a rare complication of the Hobart method for LISA. Awareness of such procedural complications is important as the use of the LISA method increases.

## Background

Respiratory distress syndrome (RDS) is a common neonatal morbidity among premature infants. There is an inverse correlation between RDS risk and gestational age, with the incidence among newborns younger than 28 weeks being as high as 93% [1]. Intratracheal instillation of surfactant is often required if initial noninvasive respiratory support fails in newborns with RDS. The usual surfactant delivery method involves endotracheal intubation and surfactant via an endotracheal tube (ET) followed by a period of mechanical ventilation. Recognizing the potential harms of routine mechanical ventilation following surfactant delivery, clinicians have adopted the technique commonly referred to as the INSURE (INtubation-SURfactant-Extubation) technique [2], which aims for rapid extubation immediately following surfactant delivery via the ET to avoid the detrimental effects of ongoing ventilation. However, the difficulty in using this technique is often the failure to rapidly extubate [3, 4], which may be related to the administration of sedatives for the procedure.

In an attempt to completely avoid the use of positive pressure ventilation for surfactant administration, several recent studies have evaluated alternative methods for surfactant delivery that are considered less invasive and allow surfactant delivery through a thin catheter placed within the trachea in a spontaneously breathing infant. These techniques have been variably called LISA (less invasive surfactant administration) and MIST (minimally invasive surfactant therapy), and they involve surfactant replacement therapy without intubation. Evidence obtained in the last decade shows that compared with INSURE or routine intubation followed by mechanical ventilation, LISA is associated with decreased days on mechanical ventilation, bronchopulmonary dysplasia rates, intraventricular hemorrhage, and mortality [5–8]. Increasingly, as scientific evidence continues to accumulate, neonatal intensive care units (NICUs) are adopting this newer method of surfactant delivery utilizing a variety of different techniques and catheters in varying patient populations. The literature regarding procedural details, ideal equipment and procedural adverse effects is scarce. Here, we describe a case of a newborn who developed airway injury leading to pneumomediastinum as a procedural complication of LISA.

## Case presentation

A female infant was born at 27 weeks of gestation to a 33-year-old, Gravida 8, Para 5 mother. The pregnancy was complicated by subchorionic hemorrhage at 11 weeks, rupture of amniotic membranes at 24 weeks gestation, and intrauterine growth restriction with absent end-diastolic flow of the umbilical artery. Doppler scans and a poor biophysical profile (2/8) at 27 weeks highlighted the need for an urgent cesarean section.

The patient received a complete two-dose course of betamethasone at 24 weeks and a single rescue dose before the C-section at 27 weeks. The birth weight was 680 g (13th percentile), and the head circumference was 22 cm (6th percentile). The infant received delayed cord clamping for 1 min, and her 5-min Apgar score was 8. Noninvasive intermittent positive pressure (NIPPV) was initiated in the delivery room, which was continued on admission to the NICU.

**Fig. 1:**
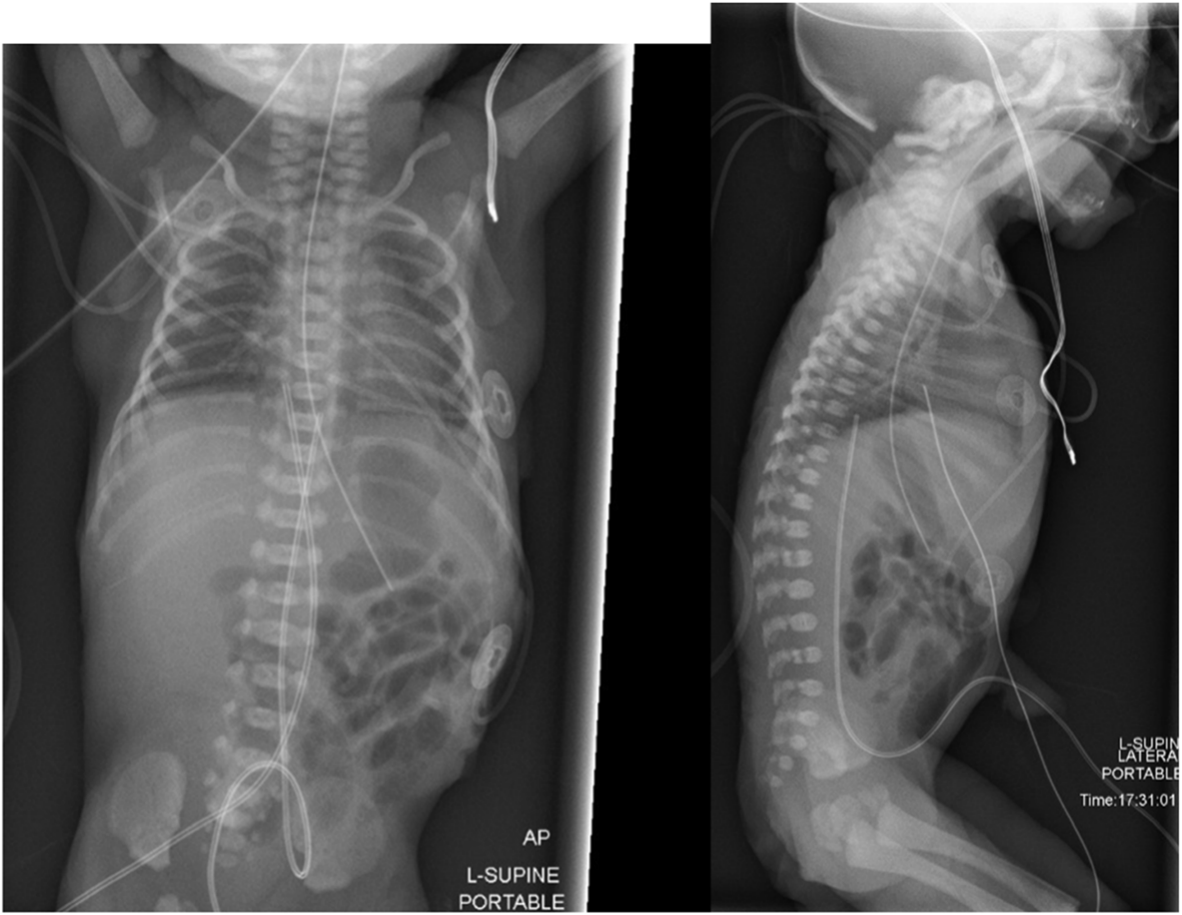
Initial AP and lateral chest X-ray showing radiographic features of RDS

A chest X-ray showed radiographic features of RDS (Fig. 1). There was an increase in the oxygen and ventilatory requirements. The infant met the institutional criteria for surfactant delivery via LISA. In our institute, the surfactant is administered via the “Hobart method”, which utilizes a semirigid 16-G catheter that is placed into the trachea without the use of Magill forceps. Bovine lipid extract surfactant (BLES®, 5 ml/kg) was successfully instilled.

The catheter was prepared by removing the introducer needle and marking the desired estimated depth. The catheter tip was slightly curved to mimic the natural airway anatomy to facilitate the insertion past the vocal cords. The infant was premedicated with atropine and fentanyl.

The procedure was performed by a neonatal physician trainee who was certified to perform LISA. The vocal cords could easily be visualized using a laryngoscope. Despite easy visualization, an attempt to insert the catheter between the vocal cords to the desired depth failed due to unexpected resistance. Given that the angiocath tip was passed beyond the vocal cords, a decision was made to start the instillation of surfactant. However, resistance was encountered during instillation, which prevented the administration of surfactant. At this point, a second visualization with the laryngoscope was conducted that confirmed that the catheter was through the vocal cords. The angiocath was then withdrawn by 0.5 cm.

A second attempt to deliver surfactant was performed, and this time, it was successfully instilled without any resistance. Subtle subcutaneous neck swelling was noted after the procedure, vital signs were normal, and no bleeding or clinical deterioration was observed. The orogastric tube was aspirated, and no surfactant was retrieved, thereby confirming that there was no inadvertent intragastric delivery of surfactant. The oxygen could be weaned down from 30 to 21% by 45 min after the procedure, and the ventilatory parameters were weaned.

**Fig. 2:**
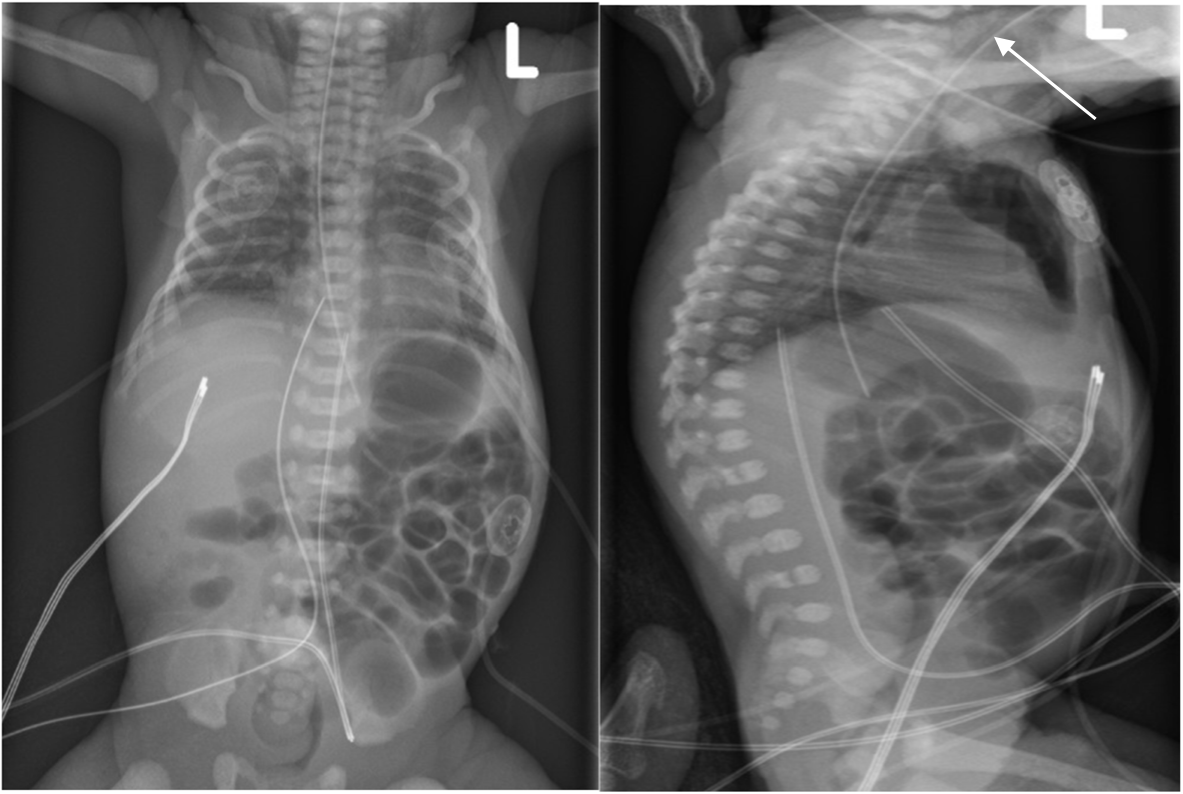
AP and lateral chest X-ray demonstrating pneumomediastinum with tracking into the soft tissue in the neck

Three hours after the procedure, the infant became hypotensive. A pneumothorax was suspected, so chest X-ray was ordered and revealed a pneumomediastinum (Fig. 2). The air leak was localized to the anterior mediastinum, with a linear radiolucency showing a tract to the anterior aspect of the trachea close to the level of the vocal cords, suggesting a high tracheal perforation. Air tracking up to the soft tissue in the neck region was noted, suggesting subcutaneous emphysema.

The infant required prompt management of hypotension, which was addressed with a fluid bolus of normal saline, and needed initiation of dopamine that was ultimately weaned off 8 h later. The pneumomediastinum was conservatively managed with close monitoring of the clinical condition and switching from NIPPV to nasal continuous positive airway pressure (nCPAP) and decreasing positive end-expiratory pressure (PEEP).

The infant remained stable from a respiratory perspective on a low level of noninvasive support. Follow-up chest X-ray revealed a gradual resolution of the pneumomediastinum, and a complete resolution of the radiographic findings was noted by day 10 of life. There was no need for a further dose of surfactant or intubation for mechanical ventilation.

### Long-term outcomes

The newborn was transitioned from nCPAP to a high-flow nasal cannula at 32 weeks. Cranial ultrasound performed on day 3 showed a grade 2 germinal matrix hemorrhage on the right side and a periventricular hemorrhage on the left side.

Subsequent cranial ultrasound examinations demonstrated the evolution of periventricular hemorrhage into cystic lesions. The infant had a brain MRI at 32 weeks that showed similar findings. The newborn also had an episode of late-onset sepsis at 20 days of life that was successfully treated with antibiotics. Enteral nutrition was gradually established. The infant was completely off respiratory support by day 50 of life at the corrected gestational age of 34 plus 1 day and therefore did not develop bronchopulmonary dysplasia. The infant was discharged home at 40 weeks’ gestation.

## Discussion and conclusions

Thoracic air leaks are collections of air that are trapped outside of the lumen of the airway. An air leak can present as a pneumothorax, pneumomediastinum, pulmonary interstitial emphysema or subcutaneous emphysema based on the location. Pneumomediastinum occurs when air escapes from the lung into extra-alveolar spaces due to rupture of an overdistended alveolus [9]. The incidence among neonates ranges from 1 to 2%, but among ventilated neonates, it can be as high as 40% [10].

Complications of the LISA procedures published in the literature include failure at the first attempt of insertion, surfactant reflux, desaturation, and bradycardia that necessitate aborting the procedure and initiating positive pressure ventilation [11, 12]. To our knowledge, there are no reports of airway injury, perforation, or an air leak. Because LISA methods have become increasingly popular and implemented in NICUs worldwide, it is crucial for users to recognize the potential for airway injury, especially in extremely premature neonates. In the case reported, we believe that the pneumomediastinum and subcutaneous emphysema over the neck were complications arising from traumatic injury to the upper airway, leading to a small perforation of the airway caused by the angiocath.

In this particular neonate, the temporal sequence of events, absence of radiological findings consistent with an air leak immediately prior to the procedure, resistance to insertion of the catheter, inability to deliver surfactant due to resistance on the first attempt and occurrence of neck swelling immediately after the procedure are highly suggestive that the air leak was the result of a procedural complication. The radiological findings that show the lucent tract connecting the pneumomediastinum to the anterior aspect of the trachea are also highly suggestive of the proposed mechanism of injury. Injury attributed to trauma from the laryngoscope itself and not the catheter cannot be ruled out but is less likely given that it was a single attempt for intubation with no reported difficulty in visualizing the airway. A comprehensive airway evaluation by a pediatric otorhinolaryngologist and actual visualization of the site of injury would have been the diagnostic gold standard. However, as the clinical and radiological findings remained stable, we opted to pursue a more conservative approach.

Laryngotracheal perforations are a rare occurrence in neonates. Previous case reports described tracheal perforation secondary to a traumatic delivery [13] and multiple endotracheal intubation attempts [13–19]. The most common location for tracheal injury is the high anterior tracheal wall [11]. The overall mortality rate of tracheal perforation is 18–22% [19, 20]. Fragile tissue, repeated manipulation, excessive force, and hyperextension are predisposing risk factors [16, 21, 22].

A variety of thin catheters have been used for this technique off label. The most common is the flexible nasogastric tube; however, umbilical artery catheters and other soft catheters have also been used for this technique [23]. The disadvantage of using flexible catheters for surfactant administration is the need for Magill forceps to facilitate placement into the trachea. To avoid the need for Magill forceps, Dargaville et al. developed a technique called the “Hobart method” that utilizes a semirigid 16-G catheter (Angiocath, Becton Dickenson) that can be placed into the trachea without the use of Magill forceps [24]. The disadvantage of this method is the potential increased risk of puncture injury when utilizing a more rigid catheter compared to a flexible catheter. The type of catheter needs careful consideration. The insertion of a straight or inadequately curved angiocath that does not follow the natural orientation of the newborn airway anatomy may increase the chances of trauma, leading to a false tract with its sharp tip. The angiocath used in the discussed procedure has a relatively elastic property. Therefore, the catheter at the time of insertion would have straightened and lost its curvature if it would not have been bent immediately before insertion. The absence of a landmark that aids in recognizing the orientation of the curvature once introduced to the oral cavity can increase the chance of creating a false tract. In personal communication with Peter Dargaville, who developed the “Hobart method”, he did not report any previous occurrences of tracheal injury with the semirigid catheter when we inquired whether this was a known potential risk of the procedure. The Hobart method is also being utilized as the technique for LISA in the large OPTIMIST multicenter RCT led by Dr. Dargaville to evaluate its use in infants born at 25–28 weeks of gestation. In total, 486 infants are currently enrolled in the trial, and there are no reports of significant safety concerns, which suggests that tracheal injury may be a very rare complication of the procedure.

Utilizing a more flexible catheter such as the nasogastric tube or one developed specifically for surfactant administration with an enhanced design (softer, blunt tip, appropriately curved introducer and markings for depth at the tip and at the lip) tailored for LISA has the potential for increasing the safety profile of the procedure. LISA from a procedural standpoint is similar to endotracheal intubation; therefore, the use of a video laryngoscope may increase the safety and success of the procedure on the first attempt [25, 26] by facilitating visualization of the catheter to the trachea to the appropriate depth, which may minimize the risk of perforation caused by failed attempts at catheter placement. As with endotracheal intubation, proper education and hands-on simulation of performing the LISA technique for clinicians without experience with the technique are also important considerations for minimizing complications of the procedure.

Management of tracheal injuries can be either surgical or conservative. Surgical management includes end-to-end anastomosis, patch repair of the puncture site, or short-term tracheostomy [19]. A conservative approach would include endotracheal intubation and bypassing the tube distal to the injury site [27]. We elected to manage this patient conservatively while maintaining noninvasive respiratory support. The newborn remained stable from a respiratory standpoint and tolerated further weaning of the mean airway pressure to prevent further worsening of the air leak. We monitored the newborn for an increase in the size of the air leak that would lead to worsening of the clinical condition and instability necessitating securing the infant with a definitive airway. The hypotension was presumed to be secondary to the air leak, which can cause increased intrathoracic pressure and decreased filling of the ventricles, leading to decreased output, with subsequent hypotension. The neurologic findings in this patient may or may not be related to this event. Watershed zones in the brain’s periventricular regions are particularly prone to damage due to cerebral ischemia [28]. Air leaks in preterm babies are known to increase the risk of intraventricular hemorrhage. Up to 89% of very-low-birth-weight infants with pneumothorax resulting in systemic hypotension can develop grade 3 or 4 intraventricular hemorrhage [29].

We report a case where procedural difficulties were noted during the LISA method of surfactant delivery, which resulted in an airway injury and a pneumomediastinum, that underwent resolution with a conservative approach. While LISA is usually reported to be an effective and safe procedure, it is important to recognize that LISA methods of surfactant delivery involve airway manipulation, and users need to be aware of rarer complications such as upper airway injury. Resistance to insertion of catheters, including ET, nasogastric or LISA, raises the suspicion of tissue injury, and clinicians should consider halting the procedure. The choice of catheter for the procedure and the use of a video laryngoscope are all factors that may modify the risk of this adverse event occurring during the LISA procedure.

## Data Availability

Data sharing is not applicable to this article, as no datasets were generated or analyzed during the current study.

## Abbreviations

RDS: Respiratory Distress Syndrome
ET: Endotracheal Tube
INSURE: INtubation-SURfactant-Extubation
LISA: Less Invasive Surfactant Administration
MIST: Minimally Invasive Surfactant Therapy
NIPPV: Intermitted Positive Pressure
NICU: Neonatal Intensive Care Unit
PIP: Peak Inspiratory Pressure
PEEP: Positive End-expiratory Pressure
nCPAP: Nasal Continuous Positive Airway Pressure
BLES: Bovine Lipid Extract Surfactant
MRI: Magnetic Resonance Imaging
